# Cerebellar and extra-cerebellar symptoms in movement disorders

**DOI:** 10.1055/s-0045-1811728

**Published:** 2025-09-19

**Authors:** Dayany Leonel Boone, Victor Rebelo Procaci, Henrique Ballalai Ferraz, Orlando Graziani Povoas Barsottini, José Luiz Pedroso

**Affiliations:** 1 Universidade Federal de São Paulo, Escola Paulista de Medicina, Departamento de Neurologia e Neurocirurgia, São Paulo SP, Brazil.

**Keywords:** Cerebellum, Basal Ganglia, Movement Disorders, Ataxia

## Abstract

The cerebellum and basal ganglia are integrated structures of the motor system, classically viewed as separate entities with different roles. Interactions between these structures were believed to occur mainly at the cortical level. However, neuroanatomical studies have resulted in a shift in this perspective. Symptoms attributed to basal ganglia disorders may arise from aberrant cerebellar circuit activity, and, conversely, cerebellar dysfunction may manifest due to pathological changes in basal ganglia pathways. In this narrative review, we present multiple disorders of the basal ganglia and cerebellum, highlighting their intricate interactions.

## INTRODUCTION

The cerebellum and basal ganglia are integrated structures within the motor system, traditionally viewed as separate entities with distinct roles. Interactions between these structures were believed to occur mainly at the cortical level. However, neuroanatomical studies have resulted in a shift in this perspective. Evidence has emerged that the cerebellum and basal ganglia form a densely interconnected network with the cerebral cortex. 1 2

Previous studies have injected the rabies virus (RV) into specific areas of the cerebellum in monkeys and used retrograde transneuronal transport of the virus to reveal the origin of multisynaptic inputs to the injection sites. 3 4 They identified a disynaptic pathway extending from the dentate nucleus to the central lateral thalamic nucleus and subsequently to the putamen, as well as a disynaptic pathway from the subthalamic nucleus to the pontine nucleus and then to the cerebellar cortex. 3 These studies also suggested that the projections from the cerebellum to the basal ganglia were topographically organized and located in both the motor and non-motor domains of the dentate nucleus. 1

In pathological conditions, dysfunction at a single node within the network can propagate throughout the entire system, leading to widespread alterations in neural activity. Consequently, symptoms attributed to basal ganglia disorders may arise from aberrant cerebellar circuit activity, and, conversely, cerebellar dysfunction may manifest due to pathological changes in basal ganglia pathways. 1

Anatomically, there is a direct connection between the basal ganglia and the cerebellum, as well as indirect disynaptic connections via the thalamus. Thus, both structures are involved in motor learning. In the same way that the cerebellum sends projections to the substantia nigra, which regulates the release of dopamine within the basal ganglia, the subthalamic nucleus also sends disynaptic projections to the cerebellar cortex. This creates a feedback loop for motor control and reward. Cerebellar dysfunction overexcites the basal ganglia, resulting in basal ganglia dysfunction. 5

Evidence from studies in rats shows that the cerebellum can rapidly modulate the activity of neurons in the substantia nigra via an excitatory monosynaptic pathway, with each structure having specific functions. The study suggests connections from the deep cerebellar nuclei (that are composed of three primary nuclei: the dentate, the interposed, and the fastigial nucleus) to the substantia nigra and suspects a reciprocal hypothesis. 6 7

The cerebellum can generate a variety of dysfunctional spike patterns, which explains why different signatures of spike sequences in the intermediate cerebellar nuclei contribute to the diverse motor impairments associated with movement disorders. 8

In vivo neuroimaging studies in patients with adult-onset idiopathic focal cervical dystonia, Parkinson's disease (PD), and spinocerebellar ataxia type 6 (SCA6) have provided insights into the microstructural differences of the cerebellar substantia nigra in various movement disorders. 9

Various neuroimaging techniques are employed to investigate and elucidate the relationship between cerebellar abnormalities and different forms of movement disorders. These include functional magnetic resonance imaging (fMRI), fluorodeoxyglucose positron emission tomography (FDG-PET), PET blood flow analysis, diffusion tensor imaging (DTI) studies, and voxel-based morphometry (VBM). 5 Recent developments in tracking techniques, combined with state-of-the-art methodologies such as optogenetics and fiber photometry, have enabled a more thorough description of the specifics and possible roles of the pathways involved in cerebellum-basal ganglia connections. 7

In this narrative review, we discuss and present the movement disorders that represent an interface between the cerebellum and the basal ganglia. First, we list the cerebellar disorders that also exhibit basal ganglia dysfunction. Finally, we describe all movement disorders related to basal ganglia impairment that may involve the cerebellum.

## METHODS

In this narrative review, we conducted a synthesis and discussion of the current literature on the connection between the cerebellum and the basal ganglia. A non-systematic search of electronic databases, such as PubMed, Scopus, Embase, and Web of Science, yielded pertinent studies. None of the datasets utilized filters or date constraints. The search was conducted using a variety of keywords, including *basal ganglia* , *cerebellum* , *ataxia* , and *movement disorders* .

The selection of publications was based on their topical significance, with a preference given to original research sections, relevant reviews, and clinical guidelines. As a narrative review, no formal quality evaluation of the studies included was conducted.

### Cerebellar ataxias and secondary basal ganglia involvement

Spinocerebellar ataxias (SCAs) are a group of hereditary ataxias characterized by progressive cerebellar degeneration, often accompanied by movement disorders. Several SCAs are known to exhibit basal ganglia dysfunction and present with both ataxia and parkinsonism, dystonia, myoclonus, tremor, chorea, and other movement disorders. 10 Beyond SCAs, several other ataxias can present with basal ganglia involvement and movement disorders. In the next section, we present a range of disorders that present this overlap.

#### *Ataxia and Parkinsonism*

**Figure 1:**
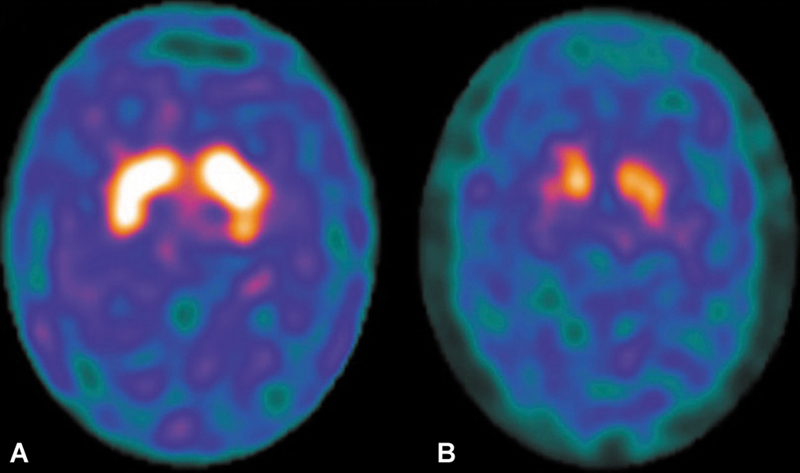
99mTc-TRODAT-1 SPECT of a healthy subject ( **A** ); decreased 99mTc-TRODAT-1 binding in all regions in a patient with spinocerebellar ataxia type 3 ( **B** ).

Parkinsonism has been described in several SCAs, most commonly in SCA2 and SCA3. Rarely, Parkinsonism has also been reported in other SCAs, including SCA1, SCA6, SCA12, SCA14, SCA17, and SCA21. 11 Patients with DOPA-responsive parkinsonism have also been reported in SCA2 and SCA3. Furthermore, several functional neuroimaging studies and pathological evaluations in SCAs have demonstrated dopaminergic pathway dysfunction, as well as changes in the substantia nigra and basal ganglia damage, particularly SCA3 ( Figure 1 ). 12 13

A previous study confirmed a significant reduction in dopamine transporter (DAT) density in the striatum, particularly in the caudate and anterior and posterior putamen of SCA3 patients. The putamen showed greater DAT loss than the caudate, as reflected by the lower putamen/caudate (P/C) ratio. These findings support the role of striatal dopamine dysfunction in the pathophysiology of SCA3. Authors have shown that striatal dopamine deficits occurred in SCA3 patients even without established parkinsonism. 14 Procaci et al. recently reported a case of SCA3 with severe presynaptic dopaminergic denervation, initially presenting with prominent tremor and later developing subtle bradykinesia and ataxia. 15

In early-onset SCA3 disease (greater than 15 years), macroscopic brain examination reveals depigmentation of the substantia nigra, as well as atrophy of the cerebellum, pons, and medulla oblongata, and atrophy of the cranial nerves (III–XII). Despite the severe degeneration of the substantia nigra, most of the affected patients do not exhibit parkinsonian features. 16

Cerebellar ataxia with sensory neuronopathy and vestibular areflexia syndrome (CANVAS) is a disorder caused by biallelic expansions in the RFC1 gene. While parkinsonism was not initially recognized as part of the syndrome, the phenotypic spectrum has since expanded, with studies demonstrating nigrostriatal dysfunction in these patients. 17

Matos et al. identified atrophy of both the basal ganglia, predominantly the caudate nuclei, and the upper brainstem through morphometric MRI analysis. 18 In contrast, a neuropathological study based on a patient with long-standing disease, revealed no significant abnormalities in the basal ganglia but demonstrated marked pallor of the substantia nigra. 19

A recent study found that in 3T MRI, SCA3 exhibited a significantly higher burden of perivascular spaces (PVS) in the basal ganglia, temporal lobe, right parietal lobe, and right cerebellum compared with healthy controls matched for sex and age. Additionally, a positive correlation was found between motor and cognitive dysfunction and greater PVS volume in the left parietal lobe and the right cerebellum. 20

Leukodystrophies can also present with a combination of ataxia and parkinsonism. For instance, in a cohort of patients with colony-stimulating factor 1 receptor (CSF1R)-related disorder, 69% exhibited ataxia and 46% had parkinsonism. 21 Neuronal intranuclear inclusion disease (NIID), a progressive neurodegenerative disease with white matter involvement, caused by a guanine-guanine-cytosine (GGC) repeat expansion in the *notch homolog 2 N-terminal-like C* ( *NOTCH2NLC* ) gene, has also been reported with parkinsonism and ataxia. 22

Hereditary spastic paraplegias (HSPs) comprise a group of genetically and phenotypically heterogeneous diseases characterized by progressive degeneration of the corticospinal tracts. The complicated forms of HSP can present with several neurological impairments, including movement disorders such as ataxia and parkinsonism. Parkinsonism has been reported in HSP patients, with some exhibiting striatal dopaminergic denervation on brain single photon emission computed tomography (SPECT). Parkinsonism seems to be more prevalent in spastic paraplegia type 7 (SPG7) patients, with reports varying between 7 and 21% of patients. The response to levodopa treatment is variable; however, some cases exhibit a good clinical response to treatment, as seen in SPG7 and SPG11. 23

#### *Ataxia and tremor*

Patients with SCA can present tremor in various forms, including rest tremor, palatal tremor, and early-onset action tremor. This early-onset form may manifest years before the cerebellar ataxia and has been described in SCA12 and SCA27. Palatal tremor was described in a patient with SCA7 and an Australian family with SCA20. Rest tremors can occur as part of a parkinsonian syndrome, as discussed above. 11

Fragile X-associated tremor/ataxia syndrome (FXTAS) is a genetically determined neurodegenerative disease which is caused by a 55 to 200 expansion of the cytosine-guanine-guanine (CGG) repeat element in the promoter region of the fragile X mental retardation 1 (FMR1) gene. Patients can present with several symptoms, such as cerebellar ataxia, cognitive decline, and psychiatric symptoms. The involvement of the cerebellum, brainstem, and basal ganglia is evident on MRI, and neuropathological findings contribute to the progression of tremors. Different types of tremors are observed in FXTAS patients; 64 to 88% exhibit intention tremor, which can be either cerebellar or essential tremor-like, as well as rest tremor in up to 12 to 30% of patients. 24

Autoimmune encephalitis can also present with combined ataxia and tremor, such as contactin-associated protein-like 2 (CASPR2)-autoantibody encephalitis and double-positive CASPR2/leucin-rich glioma inactivated (LGI1)-antibody encephalitis. 25

#### *Ataxia and dystonia*

Dystonia has been described in patients with SCA2, SCA3, SCA6, SCA7, SCA14, and SCA17. Several types of dystonia are known to occur in SCAs, from focal to generalized. 11 26 Dystonia is also associated with more severe disease and higher cytosine-adenine-guanine (CAG) repeats in some SCAs. 27

Dystonia has also been described in recessive ataxias such as Friedreich ataxia (FA), ataxia with isolated vitamin E deficiency (AVED), and ataxia-telangiectasia (A-T), among others. 28 Friedreich ataxia is the most common autosomal recessive ataxia and is caused predominantly by homozygous GAA intronic triplet repeat expansions in the *FXN* gene. The clinical picture includes progressive ataxia, dysarthria, limb weakness, sensory loss, areflexia, and extensor plantar responses. Although involuntary movements are not uncommon, they are rarely the dominant feature. Notably, in one series, 45% of patients exhibited mild upper limb dystonia, typically observed on neurological examination but often unreported by patients. 29 30

#### *Ataxia and myoclonus*

Myoclonus is a movement disorder that can originate in various areas of the nervous system, ranging from the cerebral cortex to peripheral nerves; therefore, it extends beyond the interaction between cerebellar and basal ganglia networks. Nonetheless, it is a movement disorder that can be seen in patients with cerebellar pathology, and it has been described in SCA1, SCA2, SCA3, SCA14, SCA19, 11 and recently in SCA42. 31

A wide range of acquired and genetic ataxias may present with myoclonus. Traditionally, the combination of progressive ataxia and myoclonus was referred to as Ramsay Hunt syndrome, which shares some clinical features with progressive myoclonic epilepsies. This eponym has been substituted with progressive myoclonus ataxia. 32 Among recessive ataxias, A-T, ATX-STUB1, and primary coenzyme Q10 deficiency type 4 (ATX-ADCK3) are notable for combining features of both ataxia and myoclonus. Subcortical myoclonus has been described in A-T and ATX-ADCK3, 32 whereas a case of ATX-STUB1 was suggestive of cortical myoclonus. 33

Acquired diseases that can present with myoclonus and ataxia include opsoclonus-ataxia syndrome, which can have an immune and paraneoplastic etiology, CASPR2-autoantibody encephalitis, celiac disease, multiple system atrophy, and acquired or sporadic prion diseases. 25 32

#### *Ataxia and chorea*

Chorea is mainly described in SCA17, but can occur in other SCAs, such as SCA1, SCA2, SCA3, SCA7, SCA14, and SCA27. 11 34 In recessive ataxias, chorea is commonly observed in A-T, ataxia with oculomotor apraxia type 1 (AOA1), and type 2 (AOA2). In A-T patients, extrapyramidal movement disorders tend to occur in all patients. In contrast, ataxia occurs in those with more severe disease, suggesting that the basal ganglia are more vulnerable to the pathophysiological mechanisms that underlie A-T than the cerebellum. 30 35 Chorea may begin around the second year of life as mild, fidgety movements of the hands and feet. A small number of patients may present with prominent chorea that involves the face or the entire body, often as the primary clinical feature. 30

Although cerebellar degeneration is the hallmark of A-T, evidence of basal ganglia involvement, particularly affecting dopaminergic pathways, has emerged but remains inconclusive. Animal studies have shown a marked reduction of tyrosine hydroxylase-positive neurons in the substantia nigra and decreased dopaminergic input to the striatum. At the same time, other models found no significant changes in dopamine levels or related metabolites. 36 37 38

Ataxia and chorea have also been described in acquired disorders such as paraneoplastic syndromes. The CV2/collapsin response mediator protein 5 (CRMP5) antibody can present with chorea and ataxia, which presumably result from immune-mediated damage to the striatum. 39

### Different movement disorders with basal ganglia involvement and secondary cerebellar dysfunction

The appropriate flow of voluntary movement depends on the cerebellum and the basal ganglia, each of which has a distinct function in the mechanism, either directly or indirectly. Neurotransmitters and pathways allow one system to affect another. Cerebellar symptoms may be present in certain conditions of the basal ganglia. 40

Movement disorders occur due to the involvement of the basal ganglia and their projection to the motor cortex. Over the past decade, it has been demonstrated that the basal ganglia can project not only to the thalamus but also to the cerebellum, which may result in projections to the motor cortex. Thus, activation originating from the basal ganglia has been identified as reaching the motor cortex. 1 41

Two other structures involved in this pathophysiology are the pontine peduncle nucleus (PPN) and the nucleus reticularis tegmenti pontis (NRTP), as well as their cerebellar projections. 41

Cerebellar deep brain stimulation (DBS) was first described in 1973 by Cooper et al. in patients with epilepsy. 42 Recent investigations have focused on the role of cerebellar stimulation in DBS systems for movement disorders, aiming to evaluate how cerebellar pathways influence movement regulation. Tai and Tseng (2018) 45 review various studies that utilize cerebellar DBS to treat conditions such as tremors, ataxia, dystonia, and PD, particularly in patients who have not responded to other treatments. The findings from these studies may vary based on the underlying cause, the indication for treatment, and the specific electrode target used. Cerebellar DBS in the dentate nucleus was also studied in patients with cerebral palsy who had dystonia, as assessed by the Burke-Fahn-Marsden Dystonia Rating Scale (BFMDRS). The procedure was well tolerated and resulted in significant clinical improvement in two of three patients within three months. 43

Non-invasive brain stimulation has been the subject of new studies and indications, particularly in diseases in which therapy or effective symptomatic medications are not available. 44 Additionally, ongoing research is being conducted into non-invasive methods of cerebellar stimulation, including transcranial direct current stimulation (tDCS) and repetitive transcranial magnetic stimulation (rTMS). 45

Recently, several studies have investigated the modulation of cerebellar cortical excitability in degenerative ataxias. 46 47 48 Investigators have reported that tDCS of the cerebellum modulates the firing rate of these quiescent cells in a polarity-specific manner, through a change in somatic transmembrane voltage, and to a lesser extent, affects granule cells and neurons in the deep cerebellar nuclei. 49 The rTMS has been widely used to examine corticospinal tract and motor cortex dysfunction in hereditary cerebellar ataxias, providing neurophysiological biomarkers to track disease progression over time. 48

When evaluating the effects of rTMS treatment on patients with SCA3, changes in local metabolism and the cerebellar microenvironment were induced in the cerebellum, contributing to symptom improvement in these patients. 50 Another study demonstrated the benefits of rTMS in patients with cerebellar multiple system atrophy, resulting in a significant reduction in scores on the Scale for Assessment and Rating of Ataxia (SARA) and the International Cooperative Ataxia Rating Scale (ICARS). 51

#### *Parkinsonian syndromes and the cerebellum*

Parkinsonism is defined as bradykinesia, in combination with either rest tremor, rigidity, or both. 52 Some studies suggest that the cerebellum plays a significant role in non-motor and motor symptoms. Part of the cognitive deficit observed in PD patients may be attributed to altered functional connectivity between the cerebellar vermis and other brain regions, likely related to cholinergic dysfunction. 53

A recent study has demonstrated reduced functional connectivity between the cerebellar vermis and the basal ganglia complex, which may be related to motor severity in PD patients. 54 The Enhancing NeuroImaging Genetics through Meta-Analysis—Parkinson's Disease (ENIGMA-PD) research initiative showed an inverse correlation between cerebellar regions and disease stages. Advanced stages (Hoehn and Yahr 3–5) are associated with smaller volumes of the posterior “non-motor” lobes, and the early stages (Hoehn and Yahr 1–2) with larger volumes of the anterior “motor” lobe. Therefore, the motor component of the cerebellum is more affected in the early stages, while the cerebellar “non-motor” pathways are involved in the later stages. Additionally, individuals with cognitive impairment had a smaller total cerebellar volume. 55

Functional neuroimaging studies of PD reveal a lack of correlation with disease duration, suggesting that cerebellar activity may serve as a compensatory mechanism for the dysfunctional basal ganglia, with specific subregions of the cerebellum being utilized to cope with motor demands. In addition, the cerebellum is hyperactive in patients with PD compared with healthy controls, and cognitive function in PD is associated with the more recently developed cerebellar regions. 56

An evaluation of 21 patients with postural instability and gait disturbance (PD-PIGD) revealed that these individuals had reduced volumes of cerebellar motor and non-motor areas compared with controls. In addition, during motor tasks, there was greater activation of the cognitive regions and decreased activity in motor areas, which may be due to gray matter atrophy or an attempt to compensate for the functional failure of cerebellar motor areas and basal ganglia. 57

**Figure 2:**
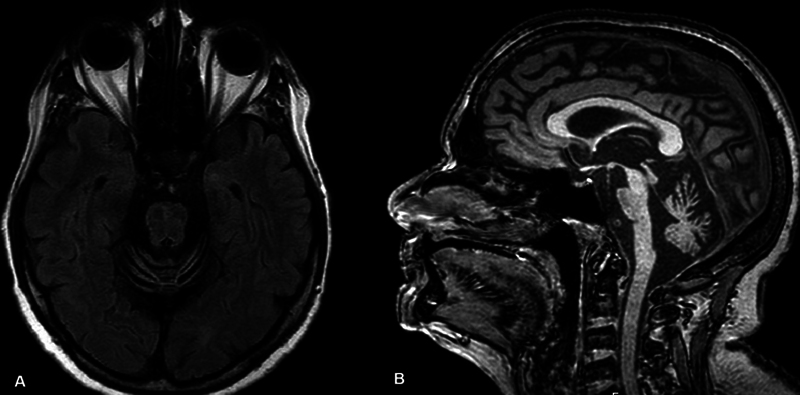
Brain magnetic resonance imaging of a patient with multiple system atrophy – cerebellar form: ( **A** ) Axial T2/fluid-attenuated inversion recovery (FLAIR) showing hyperintensity pattern in a hot cross bun configuration; ( **B** ) Sagittal T2/FLAIR showing cerebellar atrophy.

The cerebellar variant of multiple system atrophy is an α-synucleinopathy characterized by autonomic dysfunction, cerebellar ataxia, parkinsonism with poor response to levodopa, rapid progression, and a worse prognosis compared to idiopathic PD. MRI findings often include cerebellar and pontine atrophy, along with the classic ‘hot cross bun’ sign, a cruciform T2/FLAIR hyperintensity within the pons, indicative of brainstem degeneration ( Figure 2 ). 58 59

#### *Essential tremor and the cerebellum*

Essential tremor (ET) is one of the most common movement disorders, characterized by kinetic postural tremor, and has significant social and functional implications. Some studies have demonstrated bilateral cerebellar activation in the pathophysiology of this condition. Elan et al. demonstrated a reduction in some cerebellar cortical neurotransmitters in patients with ET. Additionally, patients with ET can exhibit a loss of Purkinje cells in the cerebellum. 60 61

In 2007, Louis et al. described torpedoes in ET. Torpedoes are ovoid swellings of the proximal portion of the Purkinje cell physiologically found in small numbers in the cerebellum, but when there are cerebellar lesions, their number increases, indicating a compensatory mechanism to the inflammatory stress on the Purkinje cells. The anatomopathological study of 33 brains of ET patients showed that 75.8% of them had pathological abnormalities in the cerebellum. 62

#### *Dystonia and the cerebellum*

Dystonia is a movement disorder caused by disrupted connections between the thalamus, the internal globus pallidus, the cerebral cortex, the cerebellum, and the brainstem. The cerebellum has been increasingly studied through the GABAergic Purkinje cells of the cerebellar cortex that project to the basal ganglia via the thalamus ( Figure 3 ). 63

Isolated dystonia does not exhibit neuronal loss, while mixed or complicated types typically have structural abnormalities in the cerebellum or basal ganglia. 64 Tarrano et al. had examined brain microstructure in 18 patients with myoclonic dystonia caused by a pathogenic variant in SGCE (MYC/DYT-SGCE). According to an investigation, microstructural abnormalities in the cerebellum and its afferent pathways, which transmit information from the cerebral cortex and spinal cord, were mainly responsible for the severity of dystonia in MYC/DYT-SGCE. 65 Findings from FDG-PETs showed increased metabolism within the cerebellum. 62 66 67

Historically, combined dystonia was thought to result from dysfunction in the basal ganglia; however, the basal ganglia do not function in isolation and are connected to other regions, receiving stimuli and sending information to many different brain regions, including the cerebellum. Therefore, the basal ganglia and the cerebellum are thought to overlap in the pathophysiology of dystonia. 5

Jackson et al. provided an important review of the primary genetic dystonias and the associated cerebellar changes. Patients with DYT1-TOR1A exhibit metabolic and network abnormalities in the cerebellum, as reported in several human neuroimaging studies. Increased tracer uptake is observed in the cerebellar hemisphere, which is typically associated with increased metabolic activity induced by inflammation, infection, or malignancy on FDG-PET. Additionally, DTI tractography revealed reduced connectivity between the cerebellum and thalamus. 5 68

Reduced connectivity between the cerebellum, thalamus, and cerebral cortex has also been shown in studies with *THAP1* . 62 In a mouse model of DYT12, cerebellar Purkinje cells displayed aberrant high frequency burst firing. The disrupted Purkinje cell output can drive aberrant activity in downstream motor circuits, including the basal ganglia, via a disynaptic pathway through the thalamus. These findings support the view that dystonia may result from cerebellar network dysfunction with widespread effects on motor control, reinforcing the concept of dystonia as a distributed network disorder rather than one confined to a single brain region.

#### *Myoclonus and the cerebellum*

Cerebellar involvement in myoclonus has been extensively studied, particularly in the cortical subtype. 70 Myoclonus may have various etiologies, including anatomical lesions, chemical intoxication, and physiological causes. Lesions in the dentato-rubro-olivary circuit, commonly known as the Guillain-Mollaret triangle, can cause palatal myoclonus, among other symptoms. 71

An example of cerebellar involvement associated with myoclonus is opsoclonus-myoclonus-ataxia syndrome. This rare autoimmune disease usually affects children and presents as a parainfectious or paraneoplastic syndrome with variable presentations. According to the cerebellar development hypothesis, the inhibition of omnipause neurons is disrupted when Purkinje cells in the dorsal vermis fail to inhibit the fastigial nuclei in the cerebellum. 72

#### *Huntington's disease and the cerebellum*

Cerebellar involvement in Huntington's disease (HD) is controversial. Some neuropathologic studies have shown reduced cerebellar volume, but without marked atrophy, as seen in the basal ganglia. 73 In contrast, other studies show significant cerebellar atrophy. 74

A recent study has shown an association between Purkinje cell loss in the neocerebellum and motor symptoms in HD. 75 There is a significant loss of Purkinje cells in the neocerebellum of HD cases with predominantly motor symptom profiles, but the neuropathologic grade of the striatum does not correct this. This suggests that Purkinje cell degeneration and striatal changes do not necessarily co-occur. 76 Padron-Rivera (2021) confirmed the finding of cerebellar degeneration in HD patients. 75

There is no correlation between the number of CAG repeats and cerebellar atrophy, although there may be a greater reduction in cerebellar volume in patients with more CAG repeats. 77 78 Additionally, a compensatory mechanism may exist in the cerebellar-striatal circuit in these patients. 79 In a case of a young woman with Parkinsonism who was diagnosed with HD, there was no change in cerebellar volume compared with other brain regions, even 3 years after the initial evaluation. 80 Nevertheless, descriptions of cerebellar involvement in juvenile-onset HD have been reported, including a case of a 6-year-old child with 169 CAG repeats, exhibiting various changes in the cerebellar cortex, such as significant loss of Purkinje cells, thinning of the molecular layer, and huntingtin-immunopositive intranuclear inclusions in Purkinje cells. 81

In a systematic review presented by Franklin et al., 14 anatomopathological studies showed emerging evidence of reduced cerebellar volume in HD. Eleven case reports/case series described macroscopic findings of cerebellar atrophy in HD patients, with or without cerebellar symptoms, and revealed various abnormalities of the cerebellum in pre-HD patients. Some clinical manifestations can be attributed to cerebellar degeneration, including impaired fine motor skills, dysarthria, ataxia, postural instability, and gait and stance imbalance. Maybe psychiatric and cognitive dysfunction can be worsened by cerebellar involvement. 82

In conclusion, understanding the relationship between the basal nuclei and the cerebellum is crucial for comprehending the pathophysiology of certain movement disorders, which are believed to operate in a more complex manner than the individual structures do. This is important because diseases are not isolated entities and can have mixed manifestations with different associated movement disorders. Functional neuroimaging is an ally in these cases, demonstrating dopaminergic dysfunction in ataxias and alterations in cerebellar volume and function in Parkinsonism, tremor, dystonia, and chorea.

Spinocerebellar ataxias have a higher prevalence of Parkinsonism, with some subtypes responding to levodopa (SCA 2 and SCA 3). Tremor is best described in SCA 12 and SCA 27. Dystonia may be focal or generalized. Chorea is best described in SCA 17.

Understanding the cerebellum as a structure involved in the complex pathophysiology of movement disorders, particularly PD, dystonia, and essential tremor, is crucial for identifying new therapeutic targets, especially in the context of invasive neuromodulation, such as DBS. Several trials of cerebellar DBS are already underway, showing promising results.

**NoteFigure 3:**
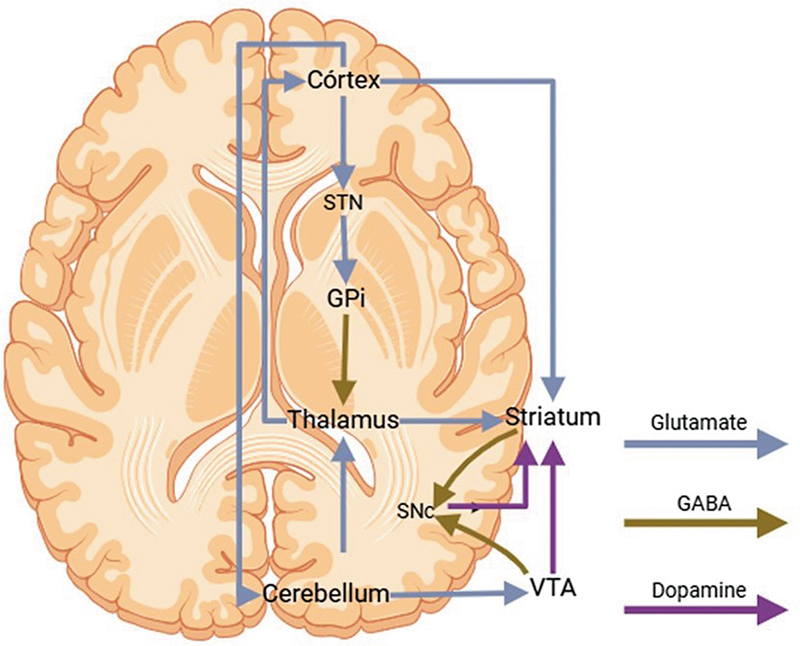
Abbreviations: SNc, substantia nigra pars compacta; GPi, globus pallidus internus; GPe, globus pallidus externus; VTA, ventral tegmental area; STN, subthalamic nucleus. : Created in https://BioRender.com . A diagrammatic representation of the brain circuits implicated in dystonia.
